# The Cross-Talks Among Bone Morphogenetic Protein (BMP) Signaling and Other Prominent Pathways Involved in Neural Differentiation

**DOI:** 10.3389/fnmol.2022.827275

**Published:** 2022-03-15

**Authors:** Asma Manzari-Tavakoli, Amirhesam Babajani, Mohammad Hadi Farjoo, Mostafa Hajinasrollah, Soheyl Bahrami, Hassan Niknejad

**Affiliations:** ^1^Department of Pharmacology, School of Medicine, Shahid Beheshti University of Medical Sciences, Tehran, Iran; ^2^Rayan Center for Neuroscience & Behavior, Department of Biology, Faculty of Science, Ferdowsi University, Mashhad, Iran; ^3^Department of Stem Cells and Developmental Biology, Cell Sciences Research Center, Royan Institute for Stem Cell Biology and Technology, ACECR, Tehran, Iran; ^4^Ludwig Boltzmann Institute for Experimental and Clinical Traumatology in AUVA Research Center, Vienna, Austria

**Keywords:** bone morphogenetic proteins (BMPs), cytokines, FGF, Sonic hedgehog (Shh), Wnt, cell therapy, nerodegenerative diseases, regenerative medicine

## Abstract

The bone morphogenetic proteins (BMPs) are a group of potent morphogens which are critical for the patterning, development, and function of the central nervous system. The appropriate function of the BMP pathway depends on its interaction with other signaling pathways involved in neural differentiation, leading to synergistic or antagonistic effects and ultimately favorable biological outcomes. These opposite or cooperative effects are observed when BMP interacts with fibroblast growth factor (FGF), cytokines, Notch, Sonic Hedgehog (Shh), and Wnt pathways to regulate the impact of BMP-induced signaling in neural differentiation. Herein, we review the cross-talk between BMP signaling and the prominent signaling pathways involved in neural differentiation, emphasizing the underlying basic molecular mechanisms regarding the process of neural differentiation. Knowing these cross-talks can help us to develop new approaches in regenerative medicine and stem cell based therapy. Recently, cell therapy has received significant attention as a promising treatment for traumatic or neurodegenerative diseases. Therefore, it is important to know the signaling pathways involved in stem cell differentiation toward neural cells. Our better insight into the cross-talk of signaling pathways during neural development would improve neural differentiation within *in vitro* tissue engineering approaches and pre-clinical practices and develop futuristic therapeutic strategies for patients with neurological disease.

## Introduction

The development of neurons is a multi-stage process that initiates early in embryogenesis that depends on several neural inducing factors (Marchal et al., 2009). The most important signaling pathways in neural differentiation include bone morphogenetic proteins (BMPs), fibroblast growth factor (FGF), cytokines, Notch, Sonic Hedgehog (Shh), and Wnt/β-catenin. BMPs are members of the transforming growth factor β (TGF-β) superfamily (Wang et al., 2014). They were discovered in the 1960s by Urist, who stated that their action in the demineralized bone matrix could persuade ectopic formation of bone (Rahman et al., 2015). The human genome contains more than 20 types of heterodimer or homodimer BMPs. According to the sequence homology of their amino acids, structures, and functions, they are divided into four separate subfamilies: (1) BMP2 and 4; (2) BMP5, 6, 7, 8a, and 8b; (3) BMP9 and 10; and (4) BMP12, 13, 14 (Bal et al., 2020). BMPs are involved in biological activities such as embryogenesis (epidermal induction and inhibiting ectodermal fate), developmental processes (cell proliferation, differentiation, and apoptosis), and also the maintenance of adult tissue homeostasis (Wang et al., 2014; Biniazan et al., 2021). BMPs genes are highly conserved which have had similar functions from fruit flies to humans. One of the best reasons for understanding the evolutionary conservation of BMP is the role of this signaling in neural induction and subsequent patterning of neuroectoderm (Mizutani and Bier, 2008). Besides, BMP family members play important roles in CNS development from neural induction to patterning of the nervous system. BMP ligands and receptors are present throughout the embryonic brain, adult brain, and spinal cord with diverse expression patterns within discrete regions of the CNS (Eixarch et al., 2018; Hart and Karimi-Abdolrezaee, 2020). BMP-2, -4, -5, -6, and -7, are expressed in the cerebral cortex, hippocampus, cerebellum, and brainstem during development (Helm et al., 2000). These BMPs are also expressed in neurons, astrocytes, oligodendrocytes, and ependymal cells of the adult brain (Hart and Karimi-Abdolrezaee, 2020). BMP-6, -12, and -14 are also expressed in midbrain dopaminergic neurons during development. BMP-9 plays a crucial role in cholinergic neurons’ specification, and BMP13 is expressed at the edge of the neural plate that participated in neural development (Helm et al., 2000; Hanel and Hensey, 2006; Meyers and Kessler, 2017). Yan et al. (2012) reported that low-level BMP-10 could be found in neurons of uninjured rats. It has been shown that transcripts for BMP-8a are present in the hippocampus while BMP-8b is expressed in hypothalamic nuclei regulating energy balance and thermogenesis (Mehler et al., 1997; Whittle et al., 2012). There is a BMP-sensitive window during neural commitment. A single BMP type can induce distinctive roles at embryonic development stages and microenvironment situations (Spatio-temporal effects). For instance, BMP 7, BMP-2, and BMP-4 induce astrocyte differentiation from neuroepithelial cells. In other conditions, these BMPs can also increase the number of neurons (Meyers and Kessler, 2017).

**Figure 1:**
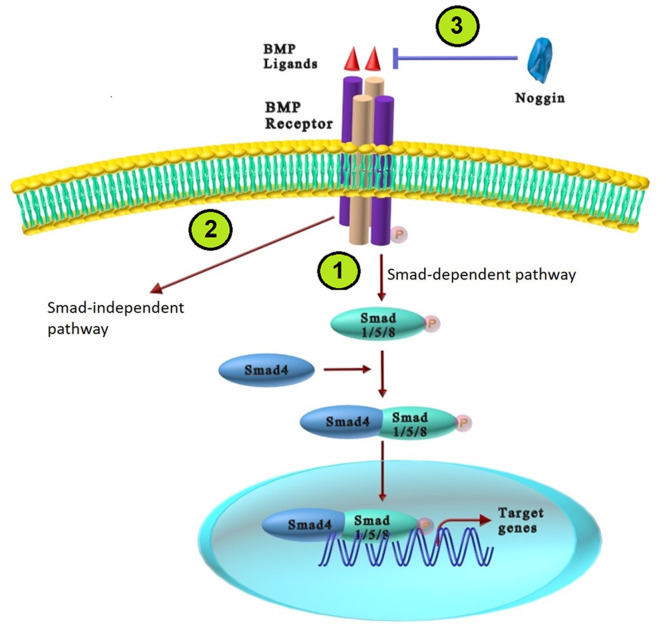
The BMP signaling pathways. **(1)** The BMP Smad-dependent (canonical) pathway: BMPs bind type II receptor and constitutively active type I receptor, which trans-phosphorylate and activate Smad1/5/8 (R-Smads). Phosphorylated Smad1/5/8 binds with Smad4, which translocate to the nucleus and regulate BMP downstream target genes. **(2)** The BMP Smad-independent (non-canonical) pathway: BMPs ligands activate the non-canonical pathway through TAK1, MKK3, and p38 mitogen activated protein kinase (MAPK). **(3)** BMP antagonists (Noggin) bind to BMPs ligands and prevent BPMs from binding to their receptors.

The BMPs exert their cellular response through two types of receptors: type I (BRIa, BRIb, ActRIa, and ActRIb) and Type II (BRII, ActRII, and ActRIIb); both types are serine and threonine protein kinases (Bandyopadhyay et al., 2013). The receptors affinities for either Type I or II receptors vary among different BMP ligands. For instance, BMP2 and BMP4 bind equally to BMPR1a and BMPR1b, but BMP7 binds less efficiently to BMPR1a. BMP6/7 can bind to BMPR1a, BMPR1b and Act-R1; and BMP14 binds to BMPR1b (Hart and Karimi-Abdolrezaee, 2020). The binding of BMP ligands to the type II receptor phosphorylates type I receptor, which consequently increases phosphorylated specific receptor-regulated Smad [a gene similar to mother against decapentaplegic (Mad) *Drosophila* gene and *Sea C. elengans*; R-Smads], including Smad1, Smad5, or Smad8. Phosphorylated R-Smads and Smad4 (a co-Smad for the BMP) modulate transcription of BMP downstream target genes by translocation into the nucleus. In addition to the Smad-dependent canonical pathway, the BMP signaling pathway plays an essential role in cell proliferation and differentiation in a Smad-independent manner (non-canonical) through TAK1, MKK3, and p38 mitogen activated protein kinase (MAPK; Miyazono et al., 2010; Bandyopadhyay et al., 2013). The BMP signaling pathway is modulated at the cellular or cytoplasmic level during neural induction. Target genes, signal transducers, and BMP ligands can inhibit this pathway at various cascade levels. BMP antagonists including Chordin, Noggin, follistatin, Gremlin, and Cerberus physically bind to BMPs and mask the critical epitopes in ligand-receptor interactions that prevent BPMs from binding to their receptors (Bandyopadhyay et al., 2013; Salazar et al., 2016). Inhibitors of BMP signaling have various binding affinities for different BMP ligands. For example, noggin, follistatin, and chordin, the first known neural inducers act by suppressing BMP signaling required for neural formation and patterning of the embryonic axis (Meyers and Kessler, 2017). Among all the physiological antagonists, Noggin is the most widely studied in the nervous system. Noggin binds with high affinity to BMPs such as BMP2, BMP4, BMP5, and BMP7; but cannot inhibit BMP6, BMP9, BMP10, and BMP11. Cerebrus also binds BMP2, BMP4, and BMP7 (Bond et al., 2012; Eixarch et al., 2018; Figure 1).

Developmental studies demonstrated that the BMPs induce either neurogenesis or glial differentiation, depending on stages of embryonic development, the source of cells, and the age of the target cells. In the initial steps of the early stages of nervous system development, inhibition of BMP is essential to create the neuroectoderm. However, higher levels of BMP signaling are vital for induction of neural crest, migration of neural cells, and spinal cord patterning at later steps. At late stages, BMP encourages astroglial commitment and prevents neuronal and oligodendroglia lineage differentiation (Gámez et al., 2013). Cell source is another indicator parameter of the role of BMPs in nervous system fate. For instance, BMPs appear to induce neuronal differentiation of ventricular zone progenitor cells, while they stimulate differentiation of subventricular zone progenitors to astrocytic lineage (Finley et al., 1999; Gomes et al., 2003). As an age parameter example, in adult brains, BMP signaling encourages the fate of astroglia while blocking the differentiation of oligodendrocytes as myeline producers and neurons (Meyers and Kessler, 2017). Recent findings have shown that neural development is more complex than only simply inhibiting BMP signaling. Therefore, considering several signaling pathways participate in the specification of neural differentiation will improve the preclinical studies for developing neural cells *in vitro*.

Differentiating neural cells from stem cells was one of the first attempts to develop appropriate nerves *in vitro*. Some studies have attempted to differentiate stem cells and progenitors into different nervous system cellular subtypes, including glial cells and neurons, which can be surgically transplanted into vertebrates to participate in nervous system regeneration (Chuang et al., 2015). Effort on the differentiation methods to prepare specific cell types, including neurons and glial cells, is one of the most critical steps toward regenerating injured neural tissues. Treatment of traumatic or neurodegenerative diseases has faced many challenges due to the low restoration capacity of the neurons (Manzari-Tavakoli et al., 2020; Biniazan et al., 2021; Jafari et al., 2021). Additionally, the shortcoming of available therapeutic approaches necessitates searching for emerging regenerative approaches (Griffin and Bradke, 2020; Leibinger et al., 2021). Cell-based therapy (CBT) is a promising therapeutic approach to regenerating damaged tissues *via* differentiating eligible cells to target cells, regulating the microenvironment of injured sites, and promoting tissue reconstruction in a paracrine manner (Babajani et al., 2020). However, applying stem cells in neural regeneration requires accurate insight into neural development pathways to recruit these signalings for inducing the differentiation of stem cells to the desired cells in the damaged tissue. This review focuses on the interaction or cross-talk between BMP signaling and other prominent neural regulatory components such as FGF, cytokines, Notch, Sonic Hedgehog (Shh), and Wnt/β-catenin. Understanding these interactions improves the prospects of stem cell biology and neuroscience and increases our future insight into stem cell application in regenerative medicine and clinical practice.

## BMP and FGF Signaling Pathway Cross-Talk in Neural Differentiation

The FGF signaling pathway regulates the brain and spinal cord neurogenesis (Oliveira et al., 2013). FGF signaling is an early and essential factor in neural differentiation during embryonic development and exerts its pivotal effect at the primitive ectoderm stage (before gastrulation; Koshida et al., 2002; Linker and Stern, 2004; Delaune et al., 2005; Stavridis et al., 2007; Cohen et al., 2010). FGFs regulate metabolic functions, tissue repair, and regeneration, frequently *via* triggering developmental signaling pathways in adult tissues (Ornitz and Itoh, 2015). The FGF protein family consists of 23 members with a crucial role through different cascades (Oliveira et al., 2013). The phylogenetic analysis suggests that FGF protein family members can be arranged into seven subfamilies containing FGF1 (FGF1, 2), FGF4 (FGF4, 5, 6), FGF7 (FGF3, 7, 10, 22), FGF8 (FGF7, 17, 18), FGF9 (FGF9, 16, 20), and FGF15/19 (FGF15/19, 21, 23) subfamily (Ornitz and Itoh, 2015). The FGF family members such as FGF1, FGF2, and FGF4; but not FGF8b, can promote neurogenesis of mouse embryonic stem cells (Oliveira et al., 2013; Chuang et al., 2015). This protein family induces its biological effect by activating FGF receptors (FGFR1–4), a subfamily of cell surface receptor tyrosine kinase (RTKs). FGF signal transduction can proceed *via* three central intracellular cascades, including RAS-MAPK, phosphatidylinositol 3-kinase (PI3K)/protein kinase B (AKT), and PLPCγ pathways (Böttcher and Niehrs, 2005; Eswarakumar et al., 2005; Chuang et al., 2015; Diez del Corral and Morales, 2017). In many experimental models, inhibiting FGF activity suppressed neural induction (Wilson et al., 2000; Koshida et al., 2002; Bertrand et al., 2003).

**Figure 2:**
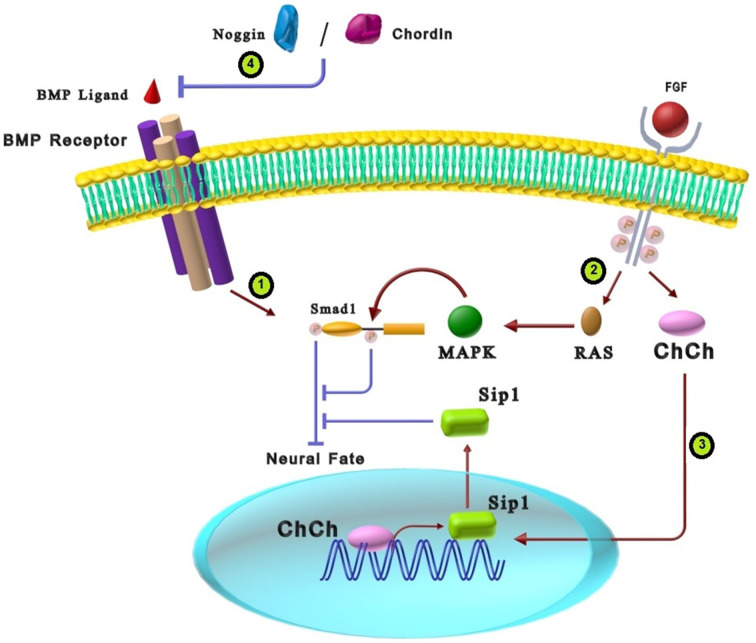
The cross-talk between BMP and FGF signaling pathways in neural differentiation. **(1)** BMP ligands activate the BMP receptors that result in Smad1 C-terminal phosphorylation and, consequently, Smad1 activation. C-terminal phosphorylated Smad1 inhibits neural fate. **(2)** Interaction of FGF ligands and tyrosine kinases receptors can activate RAS-MAPK intracellular signaling that causes phosphorylation of Smad1 linker domain. Phosphorylation of Smad1 in the linker region results in inhibition of Smad1. Inactivation of Smad1 due to FGF function induces neural fate in stem cells. **(3)** FGFs can also stimulate a zinc finger transcriptional activator, Churchill (ChCh), which increases Sip1 expression. Sip1 suppresses BMP signaling that consequently induces neural fate. **(4)** There is a strong synergy between the effects of BMP antagonists (Chordin and Noggin) and FGF in neural differentiation.

Appropriate cross-talk between BMP and FGF is mandatory for proper neural development. In this way, activation of FGF signaling synergizes with BMP inhibition to induce neural markers (Linker and Stern, 2004; Meyers and Kessler, 2017). Some researchers proposed that FGF signaling primarily exerts its effect by repressing BMPs genes or genes/proteins involved in the BMP signaling pathway (Wilson et al., 2000; Koshida et al., 2002; Pera et al., 2003; Böttcher and Niehrs, 2005; Delaune et al., 2005; Gaulden and Reiter, 2008). As shown in Figure 2, a possible molecular mechanism for the neutralizing effect of FGFs is the MAPK pathway converging on BMP signaling *via* phosphorylation of the linker domain of Smad1 *via* Ras/MAPK (Kuroda et al., 2005; Reversade et al., 2005; Fuentealba et al., 2007). The linker domain of Smad has sequences of serine/threonine residues that receive modulatory inputs from several intracellular kinase cascades, including MAPK and glycogen synthase kinase 3β (GSK3β; Sapkota et al., 2007). As previously mentioned, BMP receptors activate Smad1 through carboxy-terminal phosphorylation, whereas MAPKs catalyze inhibitory phosphorylation in the Smad1 linker region (Gaulden and Reiter, 2008; Cohen et al., 2010; Itasaki and Hoppler, 2010). Therefore, a double phosphorylation mechanism is suggested as a molecular basis for the cross-talk between BMP and FGF in neural induction (Koshida et al., 2002). This feature explains BMP and FGF signaling pathway cross-talk that prevents Smad nuclear translocation and attenuation of the BMP signaling pathway. Evaluation of neural development in chick epiblast cells showed that FGF3 induces neural fate in early epiblast cells by suppressing BMP4 and BMP7 (Wilson et al., 2000). In another study, FGF2 induced trans-differentiation of neural progenitor cells (NPCs) into oligodendrocyte progenitor cells (OPCs) *via* activating the MAPK pathway that consequently inhibits BMP signaling at the Smad1 transcription factor level (Bilican et al., 2008).

As another cross-talk between FGF and BMP, strong synergy exists between the effects of chordin and Noggin as BMP antagonists and FGF in neural differentiation. FGF pathway and BMP antagonists can attenuate Smad1 activity that results in neurogenesis induction but in different manners. While FGF can induce the Smad1 linker region’s phosphorylation *via* the MAPK pathway, BMP antagonists prevent the phosphorylation of carboxy-terminal serines in Smad1 through inhibition of BMP signaling (Figure 2; Pera et al., 2003). FGF2 works in close cooperation with Noggin to induce neural fate in developing *Xenopus* embryos (Wilson et al., 2000; Koshida et al., 2002; Bertrand et al., 2003). Additional evidence demonstrated that low amounts of FGF4 along with BMP inhibitors can trans-differentiate epidermal cells into neural cells (Marchal et al., 2009).

Neural induction in chicks also shows another cross-talk between FGFs and BMP signaling. It is demonstrated that FGFs promote a zinc finger transcriptional activator, Churchill (ChCh), which is essential for expressing Smad-interacting protein1 (Sip1) in the neural plate. As shown in Figure 2, Sip1 binds and suppresses Smad1/5 mediated activation of target genes, which in turn results in BMP signaling suppression (Böttcher and Niehrs, 2005). Taken together, cross-talk of the FGF signaling with BMP in neural differentiation is through suppressing the expression of genes involved in the BMP signaling pathway or inactivating molecules involved in the BMP signaling pathway. The crucial role of Smad1 as an interface for the integration of FGF and BMP signals is a critical clue for improving differentiation methods of stem cells.

## BMP and Cytokines Signaling Pathway Cross-Talk in Neural Differentiation

**Figure 3:**
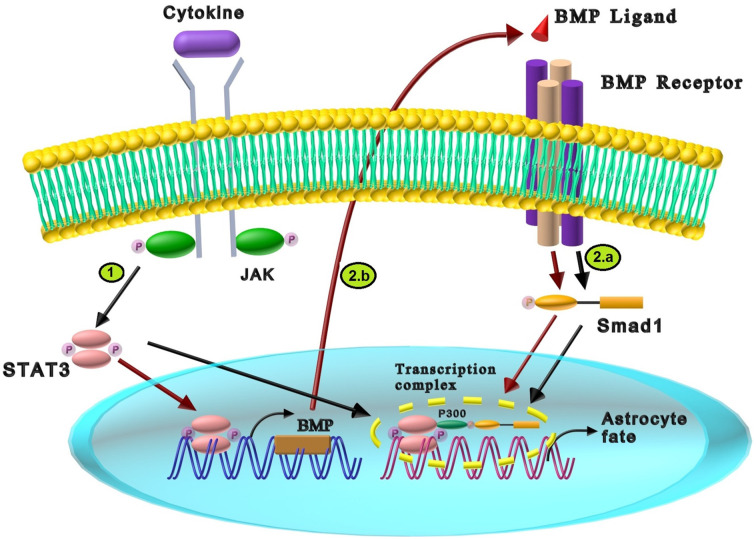
The cross-talk between BMP and cytokines signaling pathway in neural differentiation. **(1)** The interaction of cytokines and JAK components results in STAT3 phosphorylation and dimerization. Dimerized STAT3 is translocated into the nucleus, leading to astrocyte differentiation. **(2)** The synergistic effects between cytokines and BMP signaling in astrocyte differentiation are exerted through two different pathways: **(a)** LIF induces STAT3, while BMP2 activates Smad1 through their receptors. p300, a transcriptional coactivator, interacts with STAT3 and Smad1 resulting in the formation of STAT3/Smad1/p300 transcriptional complex and promotion of astrocyte differentiation (Black arrows). **(b)** Cytokines (LIF), can create a booster para-regulatory pathway in which activated STAT3 increases the expression of BMP2. The presence of BMP2 increases the activated Smad1 which induces astrogliogenesis (Red arrows).

Interleukin-6 (IL-6) family members such as leukemia inhibitory factor (LIF), cardiotrophin-1 (CT-1), and ciliary neurotrophic factor (CNTF) are among the central neuropoietic cytokines which regulate the proliferation, development, and differentiation of neural stem cells in different stages of neural development by recruiting the Janus kinase (JAK)-signal transducer and activator of transcription (STAT) signaling pathway (Bauer, 2009; Nicolas et al., 2013; Siveen et al., 2014; Borsini et al., 2015). The JAK-STAT intracellular cascade initiates by cytokine-mediated activation of cell membrane receptors. Cytokines induce dimerization of glycoprotein130 (gp130), a multichain receptor complex on the cell membrane, followed by JAK activation. Subsequently, JAK phosphorylates the tyrosines of STAT monomers in the cytoplasm, which leads to STATs dimerization. The pSTAT dimer translocates into the nucleus and regulates the transcription of various target genes (Figure 3; Nicolas et al., 2013).

Astrocyte differentiation greatly depends on STAT3 activation and its binding to the promoter region of the target gene (Kim et al., 2010; Wang et al., 2015). In embryonic cortical precursor cells, activation of the CNTF receptor triggers the differentiation of precursor cells into astrocytes by activating JAK1, STAT1, and STAT3. It has been shown that embryonic neurogenesis and neurite outgrowth were enhanced in STAT3 knock-down mice, while astrogliogenesis was inhibited (Bonni et al., 1997). The additional factors and pathways seem to regulate astrogliogenesis. Almost all regulatory mechanisms of astrocyte differentiation, such as BMPs, bFGF, and Notch collaborate with the JAK-STAT pathway to modulate the astrogliogenic process (He et al., 2005).

The cross-talk between IL-6 family members and BMPs induces astrocyte development in primary fetal neural progenitor and neuroepithelial cells in two ways: (1) formation of STAT3/Smad1/p300 transcriptional complex; and (2) a para-regulatory pathway between LIF and BMP2. In the first mechanism, LIF activates STAT3, while BMP2 triggers Smad1 through their specific receptors. As shown in Figure 3, the collaborative signaling of LIF and BMP2 requires bridging between STAT3 and Smad1, which is mediated by histone acetyltransferase p300, a transcriptional coactivator. p300 interacts with STAT3 and Smad1 through its own amino and carboxyl terminus portions, respectively, resulting in STAT3/Smad1/p300 transcriptional complex activation and astrocytes creation. Therefore, LIF and BMP2 cooperatively promote astrocyte differentiation (Nakashima et al., 1999a, b). It has been shown that cross-talk of BMP7 with LIF and IL-6 induce neuroepithelial cell differentiation to astrocytes. Similar to BMP2, BMP7 induces the formation of STAT3/Smad1/p300 transcription complex by p300-mediated bridging (Yanagisawa et al., 2001). It was reported that CT-1 was expressed in mouse fetal neuroepithelial cells, which synergistically induced astrocyte differentiation with BMP-2. Since CT-1 activates STAT3 (similar to LIF), it is probable that CT-1 acts in a similar mechanism as LIF (Ochiai et al., 2001). In the second mechanism, LIF forms a booster para-regulatory pathway in which activation of LIF/STAT3 induces the expression of BMP2 and the following Smad1 activation that encourages astrogliogenesis. Besides, this LIF-mediated upregulation of BMP2 forms the molecular basis for cross-talk between cytokines and BMPs signaling pathways, which enhances the formation of the astrogliogenic transcription factor complex STAT3/Smad1/p300 (Figure 3; Fukuda et al., 2007).

It seems that all of the IL-6 family cytokines, which activate STAT3, potentially cooperate with BMPs to enhance astrocyte differentiation. The complex creation of Smad1 and STAT3 linked by p300 and para-regulatory pathways are suggested astrocyte differentiation mechanisms.

## BMP Signaling Cross-Talk with Notch Signaling Pathway in Neural Differentiation

**Figure 4:**
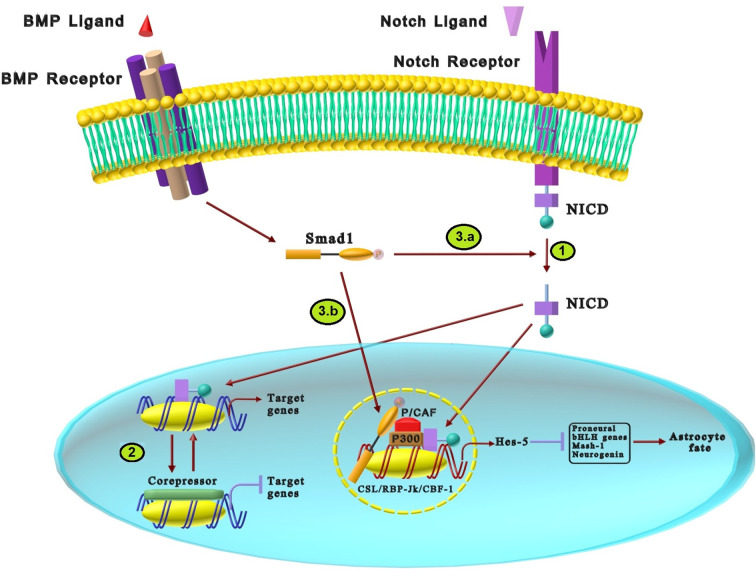
The cross-talk between BMP and Notch signaling pathways in neural differentiation. **(1)** The interaction of Notch ligands and single-pass transmembrane receptors results in the proteolytic breakdown and release of the NICD. Translocation of NICD into cell nucleus activates the CSL/RBP-Jk/CBF-1 transcription complex that increases the expression of Notch target genes. **(2)** In the absence of NICD, the co-repressor binds to the CSL transcription factor and inhibits the transcription of target genes, while the interaction of NICD with CSL promotes the transcription of target genes. **(3)** The cross-talk between BMP and Notch signaling in astrocytes differentiation is mediated *via* two distinctive ways: **(a)** BMP2 stimulation activates Smad1 that promotes the recruiting of NICD intracellular domain. Activated NICD increases activation of the Hes-5 gene promoter. **(3.b)** BMP2 promotes Smad1 to interact efficiently with NICD in presence of p300 and P/CAF that forms a transcription complex. This complex upregulates Hes-5, which inhibits proneural bHLH transcription factors such as Mash-1 and neurogenin and increases astrocyte differentiation.

Notch signaling is critical for regulating polarity and glial differentiation during early nervous system development (Chuang et al., 2015; Borggrefe et al., 2016). Notch signaling induces its effects through two distinctive pathways: canonical and non-canonical (Layden and Martindale, 2014). Because there is no acceptable data on the cross-talk of the non-canonical pathway with BMP in the nervous system development, only the canonical pathway is discussed. The Notch canonical pathway has five ligands (Delta1, 3, 4, and Jagged 1, 2) that interact with four different receptors (Notch1–4; Andersson et al., 2011; Noisa et al., 2014; Zhao et al., 2014). As shown in Figure 4, the binding of ligands, including Delta and Jagged, to the single-pass transmembrane receptor leads to receptor proteolytic breakdown and release of the Notch intracellular domain (NICD). The released domain translocates into the nucleus, activating the transcription complex named CSL/RBP-Jk/CBF-1. In the absence of NICD, CSL inhibits transcription *via* binding to the co-repressor complex, while the interaction of CSL and NICD induces basic helix–loop–helix (bHLH) transcription factors such as hairy and enhancer-of-split (Hes), Hey, Herp target genes (Figure 4; Miyazono et al., 2005; Borggrefe et al., 2016). bHLH is a structural motif on some proteins which is one of the most prominent superfamilies of dimerizing transcription factors. Studies have shown the diverse functions of bHLH in developmental processes in the nervous system, sex determination, and muscles. Their function can be highly regulated through two mechanisms: (1) dimerizing the subunits; and (2) forming heterodimers with proteins containing bHLH structure (Jones, 2004). Thus, Hes, Hey, and Herp genes can regulate the bHLH transcription factors that are necessary to induce downstream effects of Notch (Guo and Wang, 2009). The members of the bHLH family can form homodimer and heterodimer complexes which may cause neurogenesis or gliogenesis fate (Iso et al., 2003). Hes and Hey genes are the most prominent Notch downstream targets in vertebrates. Hes1, Hes3, and Hes5, as members of the Hes family, are expressed by embryonic neural stem cells that promote the generation of astrocytes and inhibit premature neuronal differentiation by suppressing proneural bHLH gene expressions such as Mash1, neurogenin 2, and Math3 (Kageyama et al., 2006; Weber et al., 2014). It has been reported that Hes1 and Hes5 inhibit neurogenesis and persuade Muller glial differentiation in the retina (Nakashima et al., 2001). As another Notch downstream target, three Hey transcription factors: Hey1, Hey2, and HeyL have been known. Hey1 and Hey2 negatively regulate neuronal bHLH genes like Mash1 and Math3 and promote the maintenance of neural precursor cells. Satow et al. (2001) has demonstrated that over-expression of Hey2 promotes gliogenesis while inhibiting neuronal development (Weber et al., 2014).

There is convincing evidence that Notch and BMP signaling interact to regulate the neural development process (Blokzijl et al., 2003; Borggrefe et al., 2016). Some studies have reported that BMP induces Notch-related target genes such as Hes and Hey family proteins in various cells, including neuroepithelial and epithelial cells, suggesting possible cross-talk between these developmental pathways (Weber et al., 2014). Nakashima et al. (2001) demonstrated that anti-neurogenic effects of BMP2 were achieved by expressing HES-5 genes in the neuroepithelial cells that inhibit proneural bHLH transcription factors such as Mash-1 and neurogenin, which subsequently leads to astrocyte differentiation. Since the transcription of HES-5 is induced by Notch as well as BMP2, there might be a signaling cross-talk between the Notch and BMP signaling pathways (Nakashima et al., 2001). Along with previous results, studies attempted to discover the underlying mechanisms of cross-talk between Notch and BMP for inhibiting neuronal fate and encouraging astrocyte differentiation. As shown in Figure 4, BMP2 can enhance Notch-induced transcriptional activation of Hes-5 in mouse neuroepithelial cells. BMP2 stimulation increases the recruiting of the NICD intracellular domain, which results in enhanced activation of the Hes-5 gene promoter (Figure 4). Additionally, BMP2 activates Smad1 that can only interact efficiently with NICD in the presence of both p300 and P/CAF to form a transcription complex. The formation of this complex triggers BMP2-mediated enhancement of Notch-induced Hes-5 expression. These interactions may suggest a novel functional cross-talk between Notch signaling and BMP signaling (Takizawa et al., 2003). Besides, some factors such as Zinc finger protein 423 (ZFP423) boost the synergistic interaction between BMP and Notch signaling by inducing synergistic interaction between NICD and the SMAD complex that causes upregulation of Hes5 gene (Masserdotti et al., 2010).

There are some studies on Notch and BMP interaction in other systems. It has been shown that Notch regulates transcription of Hes1 in cooperation with Smad3 as a downstream factor of BMP signaling. Smad3 can affect the promoter regions of Hes1 *via* direct interaction with NICD (Blokzijl et al., 2003). Furthermore, Smad1 and NICD also physically interact and cooperatively activate the transcription of Hey1 (Itoh et al., 2004). Considering the role of these genes in nervous system development, Notch and BMP pathways would induce their cross-talk in a similar way to the other body systems that can be evaluated in future studies. The cross-talk between Notch and BMP is shown in Figure 4.

## BMP Signaling Cross-Talk with Shh Signaling Pathway in Neural Differentiation

**Figure 5:**
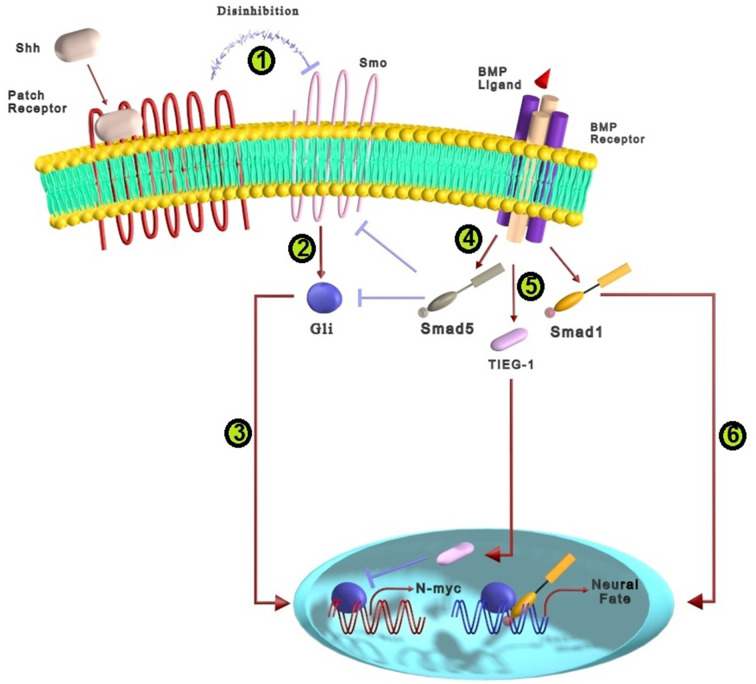
The cross-talk between BMP and Shh signaling pathways in neural differentiation. **(1)** The complex of Shh ligands and Patch receptors trigger Shh signaling pathway by preventing inhibitory effects on Smo protein located within the membrane. **(2)** Smo activates the members of zinc-finger transcription factors called the Gli family. **(3)** The translocation of Gli proteins into the nucleus increases the transcription of genes that participate in the specification of neural cells. **(4)** BMPs can prevent the effects of Shh signaling by activating Smad5, which inhibits downstream agents of Shh such as Smo and Gli1. **(5)** TIEG-1, a BMP2 target gene, can suppress Gli-mediated transcription of N-myc, a critical Shh target gene, that prevents proliferation and promotes differentiation of granule cell precursors. **(6)** Smad1 interacts with C-terminally truncated Gli proteins and form a protein complex that contains Gli proteins which lead to neural differentiation.

Sonic Hedgehog (Shh) acts as a morphogenic factor in the patterning and cell-fate specification of the central nervous system. During the development of the mammalian CNS, Shh regulates the proliferation, differentiation, and survival of neural stem/progenitor cells (Komada, 2012). Shh induces the specification of ventral neuron types and oligodendrocytes from undifferentiated neural progenitors (Agius et al., 2004). Shh acts through two different pathways: canonical and non-canonical. Canonical pathway works through Patched (Ptch) receptors/transmembrane protein smoothened (Smo)/Gli proteins (Li et al., 2021). Since there is insufficient evidence for cross-talk of the non-canonical pathway with BMP in the nervous system, only the Shh canonical pathway cross-talk with BMP is debated. As shown in Figure 5, the Shh ligand binds Ptch1/Ptch2 12-transmembrane receptors that activate their own signaling pathway by suppressing inhibitory effects on transmembrane Smo protein. Consequently, activated Smo triggers the Gli family members as zinc-finger transcription factors. Consequent translocation of Gli proteins into the nucleus results in the transcription of target genes involved in the specification of neural cells, including HNF3β, patched, Nkx2.2, and netrin-1 (Yam and Charron, 2013; Kong et al., 2015). BMPs and Shh-related proteins may be coexpressed at some cell-cell interaction sites and have conflicting activities in some neural developmental processes. It has been demonstrated that Gli members (Gli3 and Gli2 ) are co-expressed adjacent to many expression sites of BMP, including the early ventral/posterior sites of the mesoderm and the dorsal part of the neural tube (Liu et al., 1998).

Many signaling factors have been implicated in regulating dorsoventral (DV) patterning during embryonic CNS development, among which BMP and Shh signalings play essential roles. Shh factors are derived from the floor plate and notochord, while BMPs are secreted from the ectoderm and roof plate (Agius et al., 2004; Ulloa and Briscoe, 2007; Bond et al., 2012; He and Lu, 2013). It seems BMP and Shh signals have antagonistic functions in controlling cell fate along the dorsoventral axis of the neural tube. Shh has been suggested as a “ventralizing” factor that induces ventral neuronal types, whereas BMPs along with Wnts are known as “dorsalizing” factors of the neural tube (Ulloa and Briscoe, 2007; He and Lu, 2013). It has been reported that Shh signaling can suppress the dorsalizing effects of BMP signaling and induce ventral patterning of the spinal cord (Bond et al., 2012). On the other hand, exposure to high BMP levels blocks the Shh-induced ventralizition, which appears to differentiate motor neurons (Liem et al., 1995). The mechanisms that determine how DV patterning responds to BMPs or Shh depend on the cross-talk between these pathways (Bitgood and McMahon, 1995). In this regard, it has been reported that BMP signaling affects some genes that participate in Shh signaling pathway. Exposure of neural cells to BMPs block the Shh-mediated induction of HNF3β and Ptc ventral marker genes that are supposed to be induced by the direct effect of Shh signaling (Liem et al., 2000). Moreover, it has been proposed that Smads are sequestered in a protein complex that contains Gli proteins. It has been suggested that there is a physiologically relevant interaction between C-terminally truncated Gli proteins and Smads. Therefore, Shh and BMP signalings act closely in neural cell development, which may be at the level of a transcriptional regulatory complex containing both Smad and Gli proteins (Figure 5; Liu et al., 1998).

Besides the mutual effects of Shh and BMP on DV patterning, these signalings can induce single-cell specification through their cross-talk effect. It is demonstrated that Shh and BMP signaling pathways regulate oligodendrocyte progenitor’s (OPC) differentiation toward oligodendroglial and astrocytic, respectively (Wu et al., 2012). The mechanism of the antagonistic effects of BMP4 and Shh on OPC differentiation to glial cells lineage is related to differences in nuclear chromatin. Shh activates histone deacetylase1 (Hdac) and promotes oligodendrocyte differentiation by increasing the peripheral compaction of chromatins related to astrocyte generation. In contrast, BMP decreases Hdac activity and mainly triggers the transcription of genes that encourage astrocyte differentiation (Wu et al., 2012). In primary cultures, BMP2 and BMP4 can inhibit the proliferative effects of Shh *via* phosphorylation of Smad5, which allows granule cell precursors (GCPs) to exit from the cell cycle and enter the differentiation program. This inhibitory effect may be conducted through the suppressive impact of BMP on transcriptional factors of Shh such as Smo and Gli1, allowing granule neuron differentiation (Rios et al., 2004). In addition, the BMP2 target gene, TGF-β inducible early gene-1 (TIEG-1), inhibits Gli-mediated transcription of N-myc, a crucial target of Shh in GCPs, thereby preventing proliferation and promoting differentiation (Figure 5; Álvarez-Rodríguez et al., 2007). It has also been reported that Shh and BMP interaction regulates GABAergic interneuron development from dorsal telencephalic progenitors. BMPs typically limit interneuron generation by dorsal telencephalic progenitors, but Shh may promote interneuron generation by antagonizing BMP (Gulacsi and Lillien, 2003). Collectively, Shh and BMP signalings cross-talk essentially participates in appropriate DV patterning as well as differentiation of progenitor cells towards glial cell lineage and neurons.

## BMP Signaling Cross-Talk with Wnt Signaling Pathway in Neural Differentiation

Wnt family is a highly conserved signaling pathway with multiple functions in nervous system development, including neural tube formation, dorsal root ganglia neurons differentiation, and midbrain development (Kléber and Sommer, 2004; Kasai et al., 2005; Faigle and Song, 2013; Zhang et al., 2015). In nervous system development, Wnt signaling exerts its activities *via* two distinctive pathways: canonical (β-catenin dependent), or non-canonical (Inestrosa and Varela-Nallar, 2014). Currently, there is no evidence of the cross-talk of non-canonical Wnt with BMP signaling and the researchers focus on the canonical pathway.

**Figure 6:**
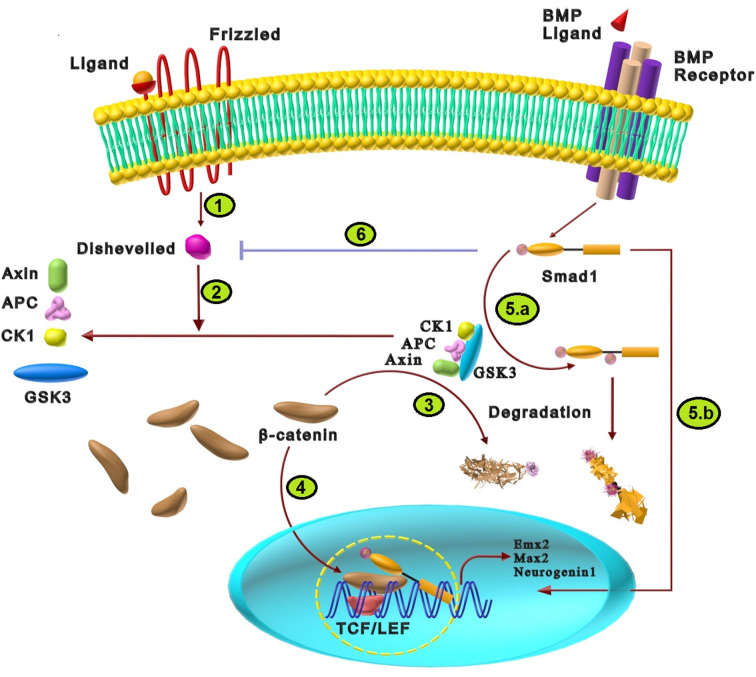
The cross-talk between BMP and Wnt signaling pathways in neural differentiation. **(1)** Wnt signaling initiation results from the interaction of Wnt ligand with the N-terminal extra-cellular cysteine-rich domain of a Frizzled (Fz) family receptor that activates disheveled. **(2)** Activated disheveled changes the destructive protein complex containing axin, APC, GSK3, and CKIα, resulting in GSK3 suppression. **(3)** Normally, the destructive complex inhibits β-catenin *via* the degrading effects of GSK3. Wnt-induced alteration of this complex increases the stability of β-catenin. **(4)** Stabilized β-catenin is translocated into the nucleus and activates TCF/LEF transcription factors that regulate the expression of Wnt target genes such as neurogenin 1, Emx2, and Max2, which induce neural fate determination. **(5)** Probable mechanisms for synergistic effects of Wnt and BMP: **(a)** Wnt inhibits GSK3 phosphorylation effects on specific sites of Smad1 in the linker region that stabilizes Smad1 and enhances BMP signaling. **(b)** Smads can directly collaborate with the β-catenin/TCF/LEF transcription complex that synergistically regulates the transcription of target genes such as neurogenin 1, Emx2, and Max2. **(6)** In the absence of any ligands, Smad1 binds and inhibits disheveled-1. The presence of BMP2 increases phosphorylated Smad1, which increases the inhibitory interaction between disheveled-1 and Smad1 that finally suppresses Wnt signaling pathway.

In the absence of Wnt ligands, the destruction complex of axin, adenomatous polyposis coli (APC), casein kinase 1(CKIα), and GSK3 is functional. In the intact form of the destruction complex, GSK3 displays enzymatic activity that leads to phosphorylation of β-catenin and resultant degradation by the proteasome system. As shown in Figure 6, binding of Wnt ligand to their cell surface receptors, including the Frizzled (Fz) family and LRP5/6, causes recruitment of axin and disheveled as cytoplasmic components of Wnt signaling. This phenomenon dissociates the destructive complex which results in inhibition of GSK-3 activity and stabilization of β-catenin. β-catenin enters the nucleus and activates T-cell factor/lymphoid enhancer-binding factor (TCF/LEF) transcription factors to regulate the expression of Wnt target genes involved in neural fate determination such as neurogenin 1, C-myc, empty spiracles homeobox (Emx)2, and Msh homeobox (Msx)2 (Guo and Wang, 2009; Ulloa and Martí, 2010; Zhang et al., 2015). Moreover, several studies have been reported that BMPs are the indirect target of wnt signaling, which inhibits BMPs expression and activates neural development (Baker et al., 1999; Wessely et al., 2001; Haegele et al., 2003).

Many studies focused on the role of Wnt signaling in adult neurogenesis. Activation of the Wnt signaling pathway enhances hippocampal neurogenesis and the differentiation of NPCs into mature neurons in Alzheimer’s disease (Oh et al., 2015). It has been shown that NeuroD1, a pro-neurogenic bHLH transcription factor, is a downstream mediator of Wnt signaling, which induces neuronal differentiation (Faigle and Song, 2013). In the other study, it has been reported that Wnt signaling enhanced cortical NPCs differentiation into neurons. The β-catenin/TCF complex directly regulates neurogenin 1, a gene that participates in cortical neuronal differentiation (Hirabayashi et al., 2004). Besides substantial evidence for the role of Wnt/β-catenin in controlling neurogenesis by promoting neuronal differentiation, some studies have demonstrated that the Wnt/β-catenin pathway can participate in the proliferation of neural progenitor cells (Zechner et al., 2003; Faigle and Song, 2013). Therefore, it seems that β-catenin may affect proliferation, differentiation, or both, depending on the presence of other signaling cascades (Otero et al., 2004).

There is cross-talk between Wnt and BMPs signalings in nervous system development through functional implications in many processes. BMP and Wnt signalings synergistically or antagonistically interact at three levels: First, BMP and Wnt regulate ligand production crucial for creating intracellular response of these morphogens during embryonic development. Second, the occurrence of cytoplasmic interactions between components of BMP/Wnt pathways. Third, they share some target genes in the nucleus (Ille et al., 2007). Their relationship varies based on the cellular context, tissue type, and development stage. Thus, the antagonistic or synergistic relation of BMPs and Wnts signaling elements regulate patterning and cell lineage commitment (Kléber et al., 2005; Guo and Wang, 2009; Itasaki and Hoppler, 2010; Bertacchi et al., 2015).

Wnt and BMP can intricately regulate each other through synergistic signaling and feedback systems during nervous system development (Itasaki and Hoppler, 2010). As the first mechanism, the absence or presence of Wnt ligands control the cross-talk between these two signaling pathways. In the absence of Wnt ligands, GSK3 phosphorylates Smad1 at specific sites in the linker region which causes the degradation of Smad1 and consequent inhibition of BMP signaling. However, in the presence of Wnt8 ligands which interact with their receptors, GSK3 is inhibited. Consequently, the BMP receptor activates Smad1 at least a few hours longer, compared to the presence of active GSK3; thus Wnt8 can enhance the effects of BMP by inhibiting GSK3 and stabilizing Smad1 (Fuentealba et al., 2007; Itasaki and Hoppler, 2010). It has been shown that Wnt and BMP signaling collaborate by the creation of the Smad/β-catenin/Lef1 complex during neural development (Guo and Wang, 2009). Additionally, it has been reported that members of the BMP family can regulate Wnt signaling in which Smad4 directly collaborates with β-catenin/TCF/Lef and synergistically regulates transcription that forms different gradients along the rostral/caudal or medial/lateral axes of the dorsal telencephalon (Kléber and Sommer, 2004). In this way, it has been reported that transcriptional targets of Smad and TCF/LEF-binding sites were juxtaposed, indicating that the two pathways may induce cooperative activity to regulate their downstream effectors, including Emx2, Msx2, and c-myc (Feigenson et al., 2011). It has been shown that synergistic Wnt/BMP signaling activity represses the differentiation of neural crest stem cells (NCSCs) and maintains multipotency in NCSCs. It seems that the homeodomain factor Msx2 might be implicated because Msx2 expression is modulated by synergistic Wnt/BMP signaling in embryonic stem cells and inhibits differentiation of migratory cranial neural crest cells. Another transcription factor, Sox10, promotes multipotency maintenance, which is continuously expressed in NCSCs treated with Wnt1 plus BMP2 (Kléber et al., 2005).

On the other hand, Wnt and BMP signaling can antagonize different processes, such as neural and neuroepithelial cell development (Feigenson et al., 2011). For example, BMP antagonizes Wnt signaling-induced proliferation in spinal cord neuroepithelial cells and Wnt suppresses BMP-induced neuronal differentiation. Thus, reciprocal inhibitory interaction between Wnt and BMP signalings controls the equilibrium between differentiation and proliferation (Ille et al., 2007). A probable mechanism for antagonistic effects of Wnt and BMP at the cytoplasmic level is disheveled-1/Smad1 direct interaction. In the lack of external Wnt and BMP ligands, disheveled-1 binds to Smad1. In the condition of cell stimulation with both Wnt3a and BMP2, phosphorylated Smad1 is generated that further increases the interaction between disheveled-1 and Smad1. Therefore, the Wnt3a-dependent stabilization of β-catenin is weakened, which is a possible mechanism of Wnt pathway inhibition by BMP signals (Itasaki and Hoppler, 2010). Direct interaction of β-catenin with inhibitory Smad is another cross-talk way between Wnt and BMP/TGF signaling (Itasaki and Hoppler, 2010).

In addition to the agonistic and antagonistic interaction of BMP and Wnt signaling, they can act as upstream or downstream effectors for each other. Wnt signaling can act upstream of BMP and upregulate its pathway in some developmental systems, such as neurogenesis and astrogliogenesis. Conversely, BMP may upregulate and act upstream of Wnt signaling in biological functions such as neural crest delamination and dorsal/ventral patterning (Fuentealba et al., 2007). In this line, it has been reported that neurons are generated at the earlier neural stages, while most glial cells are produced later. Wnt signaling triggers astroglial differentiation through stimulation of BMPs expression in neuronal cells. Hence, the cooperation of two signalings may play a critical role in neurogenesis and gliogenesis of CNS (Kasai et al., 2005). Another study also showed that Wnt signaling upregulates BMPs that cause inhibition of oligodendroglial differentiation at the neural stem cell stage. It is indicated that both BMP4 and Wnt3a are necessary for inhibiting the specification of OPCs (Feigenson et al., 2011). Thus, the BMP signaling pathway is essential for the Wnt signaling pathway to inhibit OPC differentiation, proposing that Wnt signals reside upstream of BMP (Feigenson et al., 2011). Interchangeably, some studies have shown that BMP can be upstream of Wnt. It has been demonstrated that BMP signals act upstream of Wnt/β-catenin signals to control Olig3 expression. The transcription factor Olig3 is expressed in neural progenitor cells, essential for the proper development of dorsal interneurons. So Wnt/β-catenin and BMP signals cooperate to control dorsal neurons’ specification in the spinal cord (Zechner et al., 2007). Overall, it seems that even though BMP is the main patterning factor, Wnt signaling also takes part in the neural specification in the spinal cord, both by modulating BMP signaling and activating the expression of specific proneural genes.

## Conclusion

Several signaling pathways regulate nervous system development during different stages of fetus formation. These signaling pathways cooperate in a synergistic or antagonistic manner that forms various signaling cross-talks. BMPs pathway as a pivotal signaling in neural development interacts with other signaling, including FGF, cytokines, Notch, Shh, and Wnt/β-catenin. FGF can increase neural differentiation by inhibiting Smads, a downstream protein in BMP signaling, mainly by inserting phosphorylations in inhibitory regions of Smads. On the other hand, cytokines increase astrogenesis in a synergistic manner with BMPs that include enhancing transcription complex and/or a para-regulatory pathway. Similarly, Notch cooperates with BMPs to increase gliogenesis based on the expression pattern of bHLH family members. Besides, Shh and Wnt increase neural differentiation by affecting Smads and/or downstream proteins of BMP signaling. In view of the complex interaction among different cellular pathways, considering the cooperation mechanisms of the BMP pathway with the other signalings can improve the pre-clinical and translational experience of using stem cells as a regenerative agent in treating nervous system damages; an issue which requires further investigation in the future studies.

## Author Contributions

AM-T, AB, and HN: conceptualization. AM-T and AB: investigation and writing—original draft preparation. AM-T, AB, MF, and MH: writing—review and editing. MF: drawing figures. HN and SB: supervision. All authors contributed to the article and approved the submitted version.

## Conflict of Interest

The authors declare that the research was conducted in the absence of any commercial or financial relationships that could be construed as a potential conflict of interest.

## Publisher’s Note

All claims expressed in this article are solely those of the authors and do not necessarily represent those of their affiliated organizations, or those of the publisher, the editors and the reviewers. Any product that may be evaluated in this article, or claim that may be made by its manufacturer, is not guaranteed or endorsed by the publisher.

## Abbreviations

AKT: Protein kinase B
APC: adenomatous polyposis coli
bHLH: basic helix–loop–helix
BMPs: Bone morphogenetic proteins
CBT: Cell-based therapy
ChCh: Churchill
CKIα: Casein kinase 1
CNTF: Ciliary neurotrophic factor
CT-1: Cardiotrophin-1
Emx: empty spiracles homeobox
FGF: Fibroblast growth factor
Fz: Frizzled
GCPs: Granule cell precursors
GSK3β: Glycogen synthase kinase 3β
Hes: Hairy and enhancer-of-split
JAK: Janus kinase
LIF: Leukemia inhibitory factor
MAPK: Mitogen activated protein kinase
MS: Multiple sclerosis
Msx: Msh homeobox
NICD: Notch intracellular domain
NPCs: Neural progenitor cells
PI3K: Phosphatidylinositol 3-kinase
Ptch: Patched
RTKs: Receptor tyrosine kinase
SCI: Spinal cord injury
Shh: Sonic Hedgehog
Sip1: Smad-interacting protein1
SMAD: A gene similar to mother against decapentaplegic (Mad) *Drosophila* gene and *Sea C. elegans*
Smo: Transmembrane protein smoothened
STAT: Signal transducer and activator of transcription
TCF/LEF: T-cell factor/lymphoid enhancer-binding factor
TGF-β: transforming growth factor β
TIEG-1: TGF-β inducible early gene-1.
