# Supplementary figures and images for: The Cross-Talks Among Bone Morphogenetic Protein (BMP) Signaling and Other Prominent Pathways Involved in Neural Differentiation
Source: Front Mol Neurosci. 2022 Mar 15;15:827275. doi: 10.3389/fnmol.2022.827275 (PMC8965007; doi:10.3389/fnmol.2022.827275)

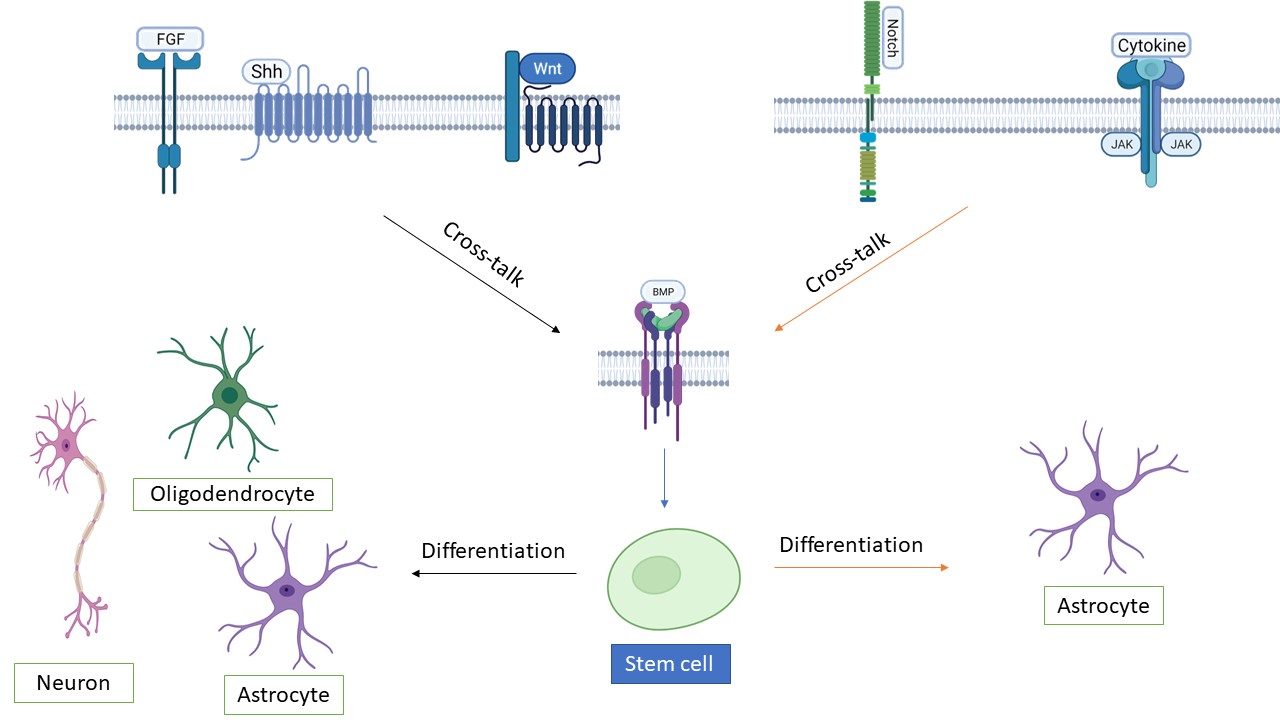

Supplement: Supplementary file 1 [file Image_1.JPEG]
